# Die traumatische Epiphysiolyse des Dens axis – ein 10-Jahres-Follow-up

**DOI:** 10.1007/s00132-025-04659-y

**Published:** 2025-05-14

**Authors:** Sara Keimling, C.-E. Heyde, P. Pieroh

**Affiliations:** Klinik für Orthopädie, Unfallchirurgie und Plastische Chirurgie, Universitätsklinikum Leipzig, Liebigstraße 26, 04103 Leipzig, Deutschland

**Keywords:** Halswirbelsäule, Kind, Konservative Therapie, Follow-up-Studie, Dorn des Axis, Cervical spine, Child, Conservative treatment, Follow-up studies, Odontoid process

## Abstract

Die Epiphysiolyse des Dens axis ist eine seltene Verletzung im Kindesalter, welche jedoch bei typischen Pathomechanismen abgeklärt werde sollte. In unserem Fall konnte die Verletzung bei einem 4‑jährigen Jungen nach Sturz auf den Nacken im initialen Röntgen nicht diagnostiziert werden. Mittels im Verlauf erfolgter MRT konnte die Diagnose gestellt und konservativ mit einer Zervikalstütze behandelt werden. Im 10-Jahres-Follow-up zeigen sich milde Beeinträchtigungen, vor allem beim langen Verweilen in einer Flexionsstellung (z. B. beim Lesen). Zudem stellt sich retrospektiv die Frage, ob eine zusätzliche Verletzung des Bandscheibenfachs C2/3 zu einer spontanen Fusion des Segmentes führte.

## Abstract (en)

Epiphysiolysis of the dens axis is a rare injury in childhood but should be reliably investigated in the presence of typical pathological mechanisms. In our case, the injury in a four-year-old boy after a fall on his neck could not be diagnosed in the initial X‑ray. The diagnosis was made by means of an MRI scan performed during the course of the case and conservative treatment with a cervical collar was provided. The 10-year follow-up showed mild impairments, especially when remaining in a flexed position for long periods (e.g. when reading). In addition, the question arises retrospectively as to whether an additional injury to the C2/3 led to spontaneous fusion of the segment.

## Einleitung

Halswirbelsäulenverletzungen im Kindes- und Jugendalter sind mit einer Inzidenz von 7/100.000 selten [6]. Die traumatische Epiphysiolyse des Dens axis gehört zu diesen Verletzungen und stellt eine klinisch relevante Verletzung im Kindesalter dar, die häufig im Zusammenhang mit Hochrisikounfällen – etwa Autounfällen, Stürzen aus großer Höhe oder Sportverletzungen – auftritt [8]. Diese Verletzung betrifft die Synchondrose zwischen dem Dens und dem Corpus axis und kann zu neurologischen Komplikationen führen, insbesondere wenn sie nicht rechtzeitig diagnostiziert wird [1]. Aufgrund der anatomischen Besonderheiten und der fortschreitenden Verknöcherung der Synchondrose im Kindesalter ist eine frühzeitige Diagnosestellung und adäquate Behandlung entscheidend, um langfristige Folgeschäden in Form von Fehlstellungen oder chronischen Schmerzsyndromen zu vermeiden [4, 13]. In der vorliegenden Arbeit werden die Diagnostik und Therapie der Epiphysiolyse des Dens axis bei einem 4‑jährigen Jungen mit einem Follow-up-Zeitraum von 10 Jahren beschrieben.

## Fallbeschreibung

Ein 4‑jähriger gesunder Junge stellte sich nach durchgeführtem Salto mit Aufprall auf den Nacken auf hartem Untergrund in der Kindernotfallaufnahme vor. Im Rahmen der körperlichen Untersuchung klagte der Patient über einen dorsalen Druckschmerz im Bereich der Halswirbelsäule (HWS), sowie Schmerzen bei Rotations- und Flexions‑/Extensionsbewegungen der HWS. Es bestanden keine sensomotorischen Defizite, keine Zeichen für eine Myelopathie und keine fixierte Fehlstellung.

**Abb. 1:**
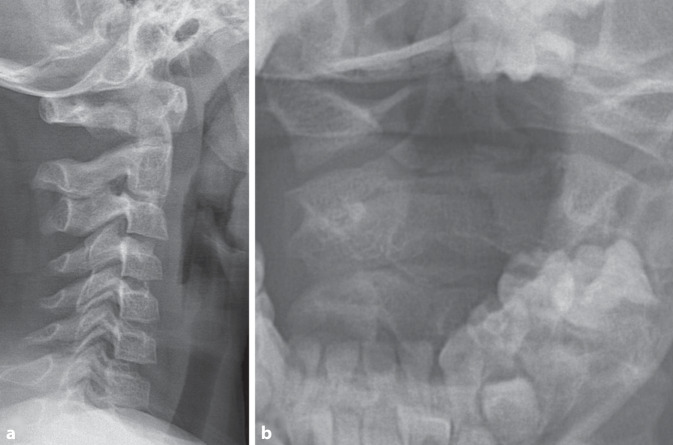
Initiale konventionelle Röntgendiagnostik (**a**) lateral und (**b**) Denszielaufnahme. Hier zeigt sich kein sicherer Frakturnachweis bei regelrechter Gelenkstellung

Das initiale Röntgen der HWS in zwei Ebenen mit Denszielaufnahme zeigte eine intakte Rahmenstruktur der Wirbelkörper, regelrechtes dorsales Alignment, keine Dislokation oder Weichteilschatten. Eine Steilstellung der HWS konnte bei anliegendem Stiffneck beobachtet werden. Die einsehbare Wachstumsfuge des Dens axis wurde als physiologisch gewertet (Abb. 1). Bei persistierender Klinik wurde eine MRT empfohlen.

Bei fehlendem Frakturnachweis erfolgte die Anlage einer weichen Zervikalstütze zur Ruhigstellung und eine bedarfsgerechte Analgesie sowie anschließende Entlassung des Patienten.

**Abb. 2:**
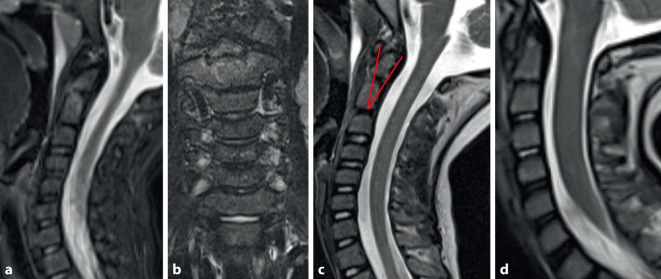
MRT („short-tau-inversion-recovery“-Sequenzen sagittal [**a,** **c**] und koronar [**b**]) 12 Tage nach Trauma – partielle Lockerung der Wachstumsfuge mit geringgradigem Ödem im Bereich des Axis (**a** und **b**), sowie dorsale Verkippung des Dens (**c**). In Abbildung (**c**) ist der „odontoid process tilting angle“ (OPTA) mit 26° (Referenz: -21,4 bis 23,3°) nach Tokunaga et al. eingezeichnet [20] (*rote Linien*). Nach Hosalkar et al. würde es sich um eine IA-Fraktur mit < 10 % Dislokation handeln [7]. Abbildung (**d**) zeigt einen Dens ohne Verkippung

Bei anhaltenden Schmerzen erfolgte 12 Tage posttraumatisch die erneute Vorstellung in der Notaufnahme mit stationärer Aufnahme zur MRT-Diagnostik ohne erneute Röntgendiagnostik. Hier fand sich eine partielle Lockerung der Wachstumsfuge zwischen Dens axis und Corpus mit geringgradig ausgeprägtem Ödem im Bereich des Dens und des Corpus (Abb. 2). Zusätzlich zeigte sich eine leichte dorsale Verkippung des Dens, sodass die Befundkonstellation für eine traumatische Epiphysiolyse auf Höhe C2 mit gering disloziertem Dens spricht. Es erfolgte eine konsequente Immobilisation in einer semirigiden Zervikalstütze für 6 Wochen und angepasster Analgesie.

**Abb. 3:**
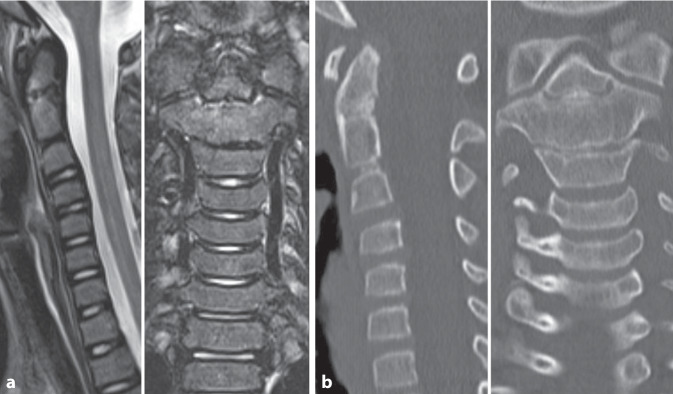
In der Verlaufs-MRT nach 3 Monaten (**a**) zeigt sich weiterhin ein ventral betontes Ödem an der Densbasis, woraufhin eine CT indiziert wurde (**b**). Hier findet sich eine progrediente Verknöcherung, ohne Anhalt auf Dislokation (**b**)

Im weiteren Verlauf erfolgten Röntgenaufnahmen direkt nach Anlage der Zervikalstütze sowie 3 Wochen, 3, 9 und 18 Monate nach Trauma ohne weitere Dislokation. Nach 1 und 3 Monaten erfolgte eine Verlaufs-MRT (Abb. 3a) sowie nach 3 Monaten ein CT der Halswirbelsäule (Abb. 3b) bei noch erhaltendem Ödem im Bereich der Densbasis.

Klinisch fanden sich Schmerzen bei Reklination aus Bauchlage, welche nachfolgend sistierten.

**Abb. 4:**
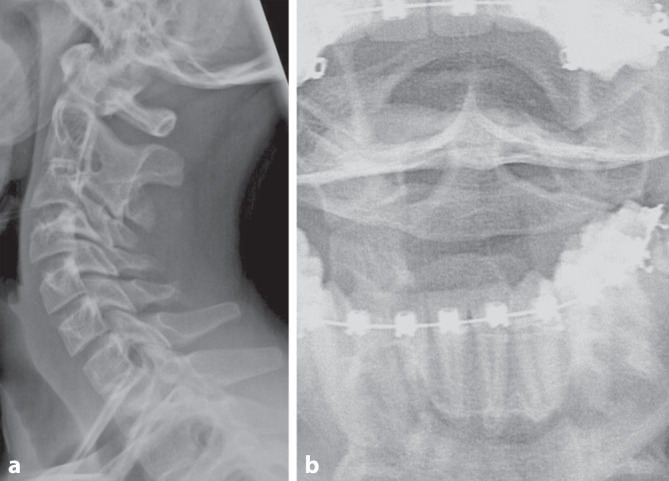
Röntgenkontrolle, seitlich (**a**) und Denszielaufnahme (**b**) nach 10 Jahren. Die Verletzung ist komplett durchbaut und es zeigt sich eine Fusion C2/3 bei regelrechtem Alignment und Gelenkstellung

Zehn Jahre nach stattgehabtem Trauma wurde der Patient aufgrund einer Osteochondrosis dissecans mit freiem Gelenkkörper im Kniegelenk vorstellig. Im Rahmen des stationären Aufenthaltes erfolgte eine Verlaufskontrolle der Epiphysiolyse des Dens axis. Dabei zeigt sich eine deutliche Verschmelzung mit Verbreiterung des Dens axis als Zeichen einer vergangenen Läsion sowie eine knöcherne Verschmelzung von C2/C3 in anatomischer Stellung (Abb. 4). Im klinischen Befund zeigt der Patient intermittierende Schmerzen im Bereich des Kiefergelenks und der Stirn, welche in physiotherapeutischer Behandlung sind. Eine Einschränkung der Sensomotorik konnte nicht festgestellt werden. Der Patient betreibt ohne Einschränkungen Leistungssport in einem Eishockeyverein.

Der Neck-Disability-Index(NDI)-Fragebogen wurde bei dem inzwischen 14-jährigen Jungen durchgeführt. Dieser erfasst Beschwerden bei alltäglichen Aktivitäten, welche von der HWS verursacht werden. Ein NDI von 0 % entspricht keiner Einschränkung, während ein NDI von 100 % einer maximalen Einschränkung entspricht [5]. In unserem Fall ergibt sich ein Score von 18 %, was mit einer milden Einschränkung einhergeht [12]. Es bestehen intermittierend leichtgradige Schmerzen vor allem bei Flexion.

## Diskussion

Die Epiphysiolyse des Dens axis ist eine seltene, jedoch wichtige differenzialdiagnostische Verletzung nach HWS-Trauma im Kindesalter. Die Verletzung tritt ausschließlich im Kindes- und Säuglingsalter auf, da die Synchondrose zwischen dem Dens axis und Corpus C2 um das 7. Lebensjahr verknöchert [4]. Das anschließende Wachstum erfolgt durch appositionelles Knochenwachstum und über den apikalen Ossifikationskern [14, 18]. Der Unfallhergang in unserer Kasuistik entspricht dabei dem typischen Pathomechanismus einer Hyperflexions‑/Hyperextensionsbewegung, welche in den meisten Falldarstellungen infolge eines Aufprallunfalls in einem Kraftfahrzeug entsteht [9]. Infolge des typischen Pathomechanismus disloziert der knöcherne Dens axis aus der Basis von C2 im Sinne einer Translations- oder Kippbewegung. Neurologische Auffälligkeiten werden dabei im Allgemeinen nur bei einer Begleitverletzung des Kopfes beschrieben [3].

In unserer Kasuistik wurde diese seltene Verletzung im Kindesalter dargestellt, welche durch die initiale röntgenologische Bildgebung nicht diagnostiziert werden konnte. Die Verletzung des jungen Patienten konnte bei Beschwerdepersistenz erst mittels MRT nachgewiesen werden. Es muss darüber diskutiert werden, ob eine MRT-Bildgebung bei Verdacht auf eine Epiphysiolyse der HWS im Kindesalter die Methode der ersten Wahl sein sollte. Vorteile ergeben sich aus der fehlenden Strahlenexposition, der höheren Sensitivität zur Detektion einer Fraktur gegenüber dem Röntgen sowie der Beurteilung einer Myelonbeteiligung. Aufgrund des höheren Wassergehalts und der erhöhten Mitoserate ist das Gewebe bei Kindern zudem strahlenempfindlicher als bei Erwachsenen, sodass es schneller zu Strahlenschäden kommt [2, 13]. Auch die früher zumeist notwendige Sedierung des Kindes kann heute durch die Einführung der Echtzeit(„real time“ [RT])-MRT umgangen werden. Hierbei konnte ein signifikant gesunkener Sedierungsbedarf von 92 % auf 55 % bei MRT-Untersuchungen bis zum Alter von 6 Jahren festgestellt werden. Durch die verkürzte Untersuchungsdauer und geringere Bewegungsartefaktanfälligkeit ist diese Untersuchungsmethode gerade für Kinder geeignet [17].

Wenn eine MRT-Diagnostik nicht zeitnah zur Verfügung steht, sollte initial eine Röntgendiagnostik in zwei Ebenen erfolgen. Zudem bietet das MRT eine mögliche Erklärung für die Fusion C2/3 in unserem Fall bei schon auffällig fehlendem Signal der Bandscheibe und einem ventralen Ödem/Flüssigkeitsverhalt (Abb. 2c und 3a). Rückblickend ist zu der dargestellten Kasuistik anzumerken, dass wir aus heutiger Sicht bei guter Stellung des Dens im Verlauf auf eine CT-Kontrolle verzichten würden.

Wird das Kind im Rahmen eines Polytraumas mit HWS-Beteiligung vorstellig, ist nach gültiger S2k-Leitlinie eine CT-Bildgebung empfohlen [19].

Die Therapie der Epiphysiolyse des Dens axis kann, wie in unserem Fall beschrieben, in den meisten Fällen konservativ durchgeführt werden.

Konservative Therapiestrategien können eine Ruhigstellung mittels Zervikalstütze oder, im Ausnahmefall, einer Halofixateur-Anlage sein. Letzteres kann jedoch aufgrund der oft eingeschränkten Compliance und des massiven Bewegungsdrangs bei den zumeist sehr jungen Patient/-innen mit Komplikationen verbunden sein [9, 11].

Durch das hohe Heilungspotenzial konnten auch bei initial größeren Fehlstellungen gute konservative Ergebnisse ohne Deformität erzielt werden.

Die operative Therapie ist Verletzungen vorbehalten, in denen die Reposition und/oder Retention nicht möglich ist oder neurologische Defizite vorliegen [20]. Hosalkar et al. empfahlen eine operative Therapie mittels C1–C2 Stabilisierung bei einer anterior-posterioren Dislokation von > 100 % [7], beispielsweise mittels Cerclagen (Draht oder Faden) oder Plattenosteosynthese beschrieben [10, 15, 16].

Nach initialer Reposition sollte unter Verlaufskontrollen eine sekundäre Dislokation und damit zeitversetzte Operationsindikation nicht übersehen werden, die Wahl der Bildgebung richtet sich nach dem individuellen Verletzungsmuster [13]. Bei sensomotorischen Einschränkungen empfehlen wir eine MRT.

## Fazit für die Praxis

- Die Epiphysiolyse des Dens axis stellt eine seltene, jedoch relevante Verletzung dar.
- Die typische Fallkonstellation ist bei einem Kindesalter von 0–7 Jahren und Hyperflexions- oder Hyperextensionsbewegungen z. B. bei Aufprallunfällen oder Stürzen auf die HWS gegeben.
- Die MRT ist der Goldstandard der initialen Diagnostik. Eine konservative Therapie ist zumeist mittels Ruhigstellung in einer semirigiden Zervikalstütze über max. 3 Monate oder nachrangig über eine Halofixateur-Anlage möglich.
- Indikationen zur operativen Versorgung sind die hochgradige Dislokation, eine persistierende Instabilität der Halswirbelsäule, neurologische Auffälligkeiten bei Beteiligung des Myelons sowie ein frustraner geschlossener Repositionsversuch oder eine sekundäre Dislokation mit anhaltender massiver Fehlstellung.

## Abbreviations

HWS: Halswirbelsäule
CT: Computertomographie
MRT: Magnetresonanztomographie
